# Pay-as-you-go liquefied petroleum gas supports sustainable clean cooking in Kenyan informal urban settlement during COVID-19 lockdown

**DOI:** 10.1016/j.apenergy.2021.116769

**Published:** 2021-06-15

**Authors:** Matthew Shupler, Mark O'Keefe, Elisa Puzzolo, Emily Nix, Rachel Anderson de Cuevas, James Mwitari, Arthur Gohole, Edna Sang, Iva Čukić, Diana Menya, Daniel Pope

**Affiliations:** a Department of Public Health, Policy and Systems, University of Liverpool, Liverpool, United Kingdom; b PayGo Energy Ltd., Nairobi, Kenya; c Global LPG Partnership (GLPGP), 654 Madison Avenue, New York, United States; d School of Public Health, Amref International University, Nairobi, Kenya; e School of Public Health, Moi University, Eldoret, Kenya

**Keywords:** Pay-as-you-go, Liquefied petroleum gas, LPG, Clean cooking fuels, COVID-19 lockdown, Informal settlement

## Abstract

Approximately 2.8 billion people rely on polluting fuels (e.g. wood, kerosene) for cooking. With affordability being a key access barrier to clean cooking fuels, such as liquefied petroleum gas (LPG), pay-as-you-go (PAYG) LPG smart meter technology may help resource-poor households adopt LPG by allowing incremental fuel payments. To understand the potential for PAYG LPG to facilitate clean cooking, objective evaluations of customers’ cooking and spending patterns are needed. This study uses novel smart meter data collected between January 2018-June 2020, spanning COVID-19 lockdown, from 426 PAYG LPG customers living in an informal settlement in Nairobi, Kenya to evaluate stove usage (e.g. cooking events/day, cooking event length). Seven semi-structured interviews were conducted in August 2020 to provide context for potential changes in cooking behaviours during lockdown. Using stove monitoring data, objective comparisons of cooking patterns are made with households using purchased 6 kg cylinder LPG in *peri*-urban Eldoret, Kenya. In Nairobi, 95% of study households continued using PAYG LPG during COVID-19 lockdown, with consumption increasing from 0.97 to 1.22 kg/capita/month. Daily cooking event frequency also increased by 60% (1.07 to 1.72 events/day). In contrast, average days/month using LPG declined by 75% during lockdown (17 to four days) among seven households purchasing 6 kg cylinder LPG in Eldoret. Interviewed customers reported benefits of PAYG LPG beyond fuel affordability, including safety, time savings and cylinder delivery. In the first study assessing PAYG LPG cooking patterns, LPG use was sustained despite a COVID-19 lockdown, illustrating how PAYG smart meter technology may help foster clean cooking access.

## Highlights

• Monthly per capita pay-as-you-go LPG consumption increased during COVID-19 lockdown.
• 95% of 301 active pay-as-you-go LPG customers maintained use during COVID-19 lockdown.
• Daily cooking events using pay-as-you-go LPG increased 60% during COVID-19 lockdown.
• Payment frequency increased 50%; single payment amount decreased 50% during lockdown.
• Average pay-as-you-go payment amount was 25% of typical full 6 kg LPG cylinder cost.

## Graphical abstract

**Figure:**
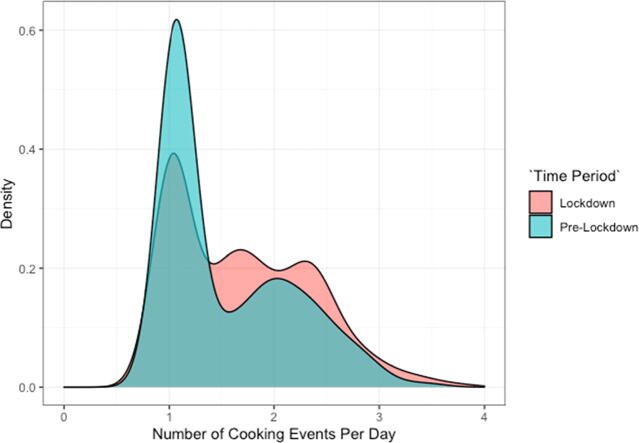

## 1 Introduction

An estimated 2.8 billion people rely on polluting fuels, including solid fuels (e.g. wood, charcoal) and kerosene, for their household energy needs [1]. Unsafe levels of fine particulate matter (PM_2.5_) generated from combustion of polluting fuels is an established risk factor for several infectious and non-communicable respiratory and cardiovascular diseases [2], [3]. Residential biomass combustion generates 25% of global emissions of black carbon (BC) [4], the dark component of particulate matter and a short-lived pollutant that has strong visible light absorption properties [4], [5], [6]. BC is estimated to have the second largest radiative forcing [5], [7] following only CO_2_ [4], [7].

Clean burning liquefied petroleum gas (LPG), although a fossil fuel, emits low levels of BC and minimal PM_2.5_ concentrations, typically meeting WHO Indoor Air Quality Guideline levels for health [8]. LPG can therefore have a neutral or ‘cooling’ effect on climate by reducing emissions of BC when it replaces use of biomass fuels for cooking in households [9], [10]. Replacing biomass cooking fuels with LPG also reduces deforestation and associated emissions of CO_2_ [11], [12], and alleviates time poverty through decreased cooking time [13]. LPG is currently used for cooking (exclusively or alongside polluting fuels [14], [15]) by over 2.5 billion people worldwide [16], especially in Latin America [17] and with rapid expansion in India [18] and Indonesia [19]. In Sub-Saharan Africa (SSA), however, 85% of the population relies on polluting cooking fuels, twice the global average (40%) [20].

### 1.1 Barriers to liquefied petroleum gas access

Polluting cooking fuels are commonly used in SSA due to their availability and low cost, or the ability to gather biomass for free from local forested areas, particularly in East Africa [11], [21]. While LPG costs are frequently lower relative to purchased biomass fuels on a per kilogram basis [22], an unreliable supply of LPG and the financial outlay required to pay upfront for pre-set units of gas (e.g. 6 kg-15 kg cylinders) under the standard branded cylinder recirculation model (BCRM) [23] is prohibitive for many [24]. Among those that can afford the initial cost of the full LPG cylinder, insecure incomes and precarious financial circumstances may prevent some households from seeking full cylinder LPG refills, leading to its unsustainable usage as a primary cooking fuel [18]. Thus, year-round affordability of LPG must be achieved to facilitate universal access to affordable, reliable, sustainable and modern household energy (Sustainable Development Goal (SDG) 7).

Despite progress in the clean cooking sector over the last two decades, population growth has exceeded gains in access to clean household energy; the International Energy Agency World Energy Outlook 2020 report states that the world is currently not on track to achieve SDG7 by 2030 [25]. The challenge of meeting SDG 7 has been further compounded by the economic fallout resulting from COVID-19 community lockdowns [10], [26]. A recent longitudinal study showed that 95% (n = 183) of households in an informal settlement in Nairobi, Kenya experienced reductions in household income during COVID-19 lockdown, and 27% of full cylinder LPG users in the community reverted to polluting cooking fuels, including kerosene (14%) or wood (13%), for cooking [27]. Thus, there is an urgent need for new innovations that can overcome the increased financial barriers caused by the COVID-19 pandemic and accelerate access to clean cooking fuels, such as LPG.

### 1.2 Pay-as-you-go liquefied petroleum gas

Pay-as-you-go (PAYG) is a consumer finance mechanism that can potentially relieve the financial barrier to sustained clean energy access. Although there is a surcharge to the fuel costs to cover equipment installation and delivery fees, PAYG LPG importantly allows consumers to purchase LPG credits in small increments (via mobile banking) [28]. Pay-as-you-go smart meter technology has already been used to provide resource-poor households in SSA with affordable access to technologies for electricity and water/sanitation [29], [30]. From a social perspective, similarities of PAYG with the concept of paying for an energy service may help explain its success [29].

PAYG LPG companies also offer LPG cylinder home deliveries that, although requiring more complex distribution logistics, can be successfully planned via real-time tracking of LPG consumption [22]. This importantly eliminates the need for customers to travel to LPG retail locations, which can be a significant LPG access barrier in some contexts [31]. PAYG can also improve LPG safety via increased transparency and efficiency of the LPG supply chain, which includes monitoring of when cylinders are running low and need refilling. This minimises the risk of illegal cylinder refilling, which may occur due to inadequate enforcement of regulatory standards in some LPG supply chains [32]. Further, as income generated in the informal or agricultural sector can vary seasonally and influence cooking fuel use [18], PAYG LPG may offer families the payment flexibility needed to maintain clean cooking during periods of reduced household income.

### 1.3 The need to evaluate the viability of pay-as-you-go

As several PAYG LPG commercial companies have penetrated the clean cooking fuel market in East Africa in recent years [22], smart meter technology may be a scalable consumer finance mechanism in SSA and one of several tools available for helping countries (e.g. Kenya, Ghana, Rwanda) achieve their ambitious targets for rapid market expansion of LPG by 2030 [33]. In Kenya, the Ministry of Petroleum and Mining has demonstrated the government’s commitment to promoting PAYG LPG as a clean energy access solution by releasing a tender for 80,000 LPG cylinder smart meters [32]. However, as PAYG LPG smart meter technology is in early piloting stages in the clean cooking fuel sector, minimal data documenting cooking patterns among customers under a PAYG LPG model is available. Hence, an evaluation of cooking behaviours among early users of the technology is critical for understanding its potential ability to foster both uptake and sustained use of LPG for clean cooking, in comparison to adoption behaviours using conventional LPG.

### 1.4 Monitoring long-term stove usage

Previous randomised controlled trails that have introduced clean cooking technologies in communities where households predominantly cooked with polluting fuels have typically been unsuccessful at achieving long-term usage of the intervention [34]. Given the extra asset costs associated with a PAYG model, monitoring of multiple years of cooking patterns under such model is needed to understand its ability to sustain use of LPG over time. Previous studies containing objective stove use measurements have typically utilized temperature sensors [35], [36], with the temperature data being dichotomized into ‘stove use’ or ‘non-use’ using advanced algorithms [37], including machine learning [38]. PAYG LPG smart meter technology has the advantage of real-time recording of the quantity (kilograms) of LPG consumed and length of time the smart meter was in use. Thus, there is an opportunity to examine PAYG LPG consumption patterns without the need for external monitoring of stove temperatures as a proxy for stove usage.

### 1.5 Study aims

This study presents an analysis of smart meter data from 426 customers of PayGo Energy in Nairobi, Kenya, one of the first PAYG LPG smart meter companies in Africa. As the first study, to the authors’ knowledge, to use PAYG LPG smart meter data to summarise consumer spending and cooking patterns, this analysis had three research goals: (1) to characterize PAYG LPG stove use and payment patterns to assess the ability of PAYG LPG to sustain clean cooking in urban areas of SSA, (2) to understand characteristics of households more likely to use PAYG LPG to inform strategies that may encourage higher rates of clean cooking and (3) to quantify the effects of the COVID-19 community lockdown and associated impacts on household income on PAYG LPG cooking behaviours.

## 2 Methods

### 2.1 Study setting and timeline

PayGo Energy was founded in 2016 to offer pay-as-you-go (PAYG) LPG with stainless steel double burner cookstoves (supplied by *Real Flame,* based in India) to residential and small commercial business customers in Mukuru kwa Reuben informal settlement in Nairobi, Kenya. PayGo Energy provides the new stove, a gas cylinder, a smart meter and fire safety equipment and includes home delivery of cylinder refills. PayGo Energy installs all equipment in the homes of new customers and provides continuous customer service support.

Mukuru kwa Reuben is a single settlement within the Mukuru cluster, which is one of the largest informal settlements in Nairobi, occupying approximately 650 acres of land in the industrial area of the city [39]. In March 2017, 50 households in Mukuru kwa Reuben were initially enrolled as PayGo Energy customers without any metering technology (see Figure S1 in Supplemental Information for timeline). In August 2017, new customers were registered with the company and supplied with metered LPG via a diaphragm (“Goldcard”) meter, capable of measuring gas consumption to the nearest 0.2 kg. Beginning in 2019, the Goldcard meters were gradually swapped to more highly calibrated ‘cylinder smart meters’ that measured customer LPG fuel usage to the nearest 0.001 kg.

### 2.2 Analysis of Pay-as-you-go LPG smart meter data

While PayGo Energy customer LPG consumption and expenditure data was available from August 2017 through June 2020, data from 2017 (before the Goldcard meters were installed) were excluded (n = 39; 8% of total sample) due to inaccurate information on LPG consumption. Customer payment and consumption data from Goldcard and smart meters in 2018–2020 was combined to increase the power of the analysis (sensitivity analyses examined data separately for each type of meter to assess robustness of results). In all analyses, separate consumption readings from the same PAYG LPG stove within the same hour were counted as a single cooking event; for example, a customer using their stove on three separate instances within the same hour and consuming 0.01 kg of gas on each occasion was considered to have a single 0.03 kg cooking event.

At time of registration for PayGo Energy equipment, new customers completed a baseline questionnaire on demographic and socioeconomic factors (the information collected from customers varied from year to year). This information was used to examine the association of various consumer characteristics with PAYG LPG consumption and expenditures.

### 2.3 COVID-19 lockdown

The study period in this analysis partially coincided with a national COVID-19 lockdown in Kenya, which was enforced beginning on March 25, 2020, two weeks after the first case of COVID-19 was detected on March 13, 2020. Two weeks after initiation of the national lockdown on April 7, 2020, a dusk-to-dawn curfew (7 pm-5 am) was implemented. The analysis of PayGo Energy smart meter data was therefore separated into pre-COVID-19 lockdown months (January 2018-February 2020), and months during COVID-19 lockdown (March-June 2020) to examine potential effects of the nationwide lockdown, and associated impacts on household income and cooking behaviours [40], on patterns of LPG usage.

### 2.4 Customer interviews

On August 18, 2020 (after the analysis period of smart meter data included in this study), a sample of seven customers (six residential and one commercial) of PayGo Energy were interviewed in-house to assess how the pandemic has affected their livelihoods; the interviewed customers were purposively selected from different areas of Mukuru kwa Reuben. The 10-minute telephonic, semi-structured interviews encompassed the following questions: (1) how has COVID-19 impacted you and your community?, (2) have you changed your spending on LPG during COVID-19 and why?, (3) do you cook with other fuels in addition to PAYG LPG? and (4) have your cooking fuel choices changed during COVID-19? The interviews were conducted in Kiswahili by two local staff members employed by PayGo Energy. After translation into English by native speakers of Kiswahili, key findings from the interviews were independently identified following the process of thematic analysis [41] in computer-assisted qualitative data analysis software (ATLAS.ti Scientific Software Development GmbH). The results of the interviews were integrated with quantitative data to provide context for PAYG LPG cooking patterns before and during COVID-19 lockdown.

### 2.5 Stove use monitoring data in Eldoret, Kenya

In a separate study, stove use monitoring (SUM) data was collected from a sample of 23 households in Eldoret, a *peri*-urban town five hours drive away from Nairobi in Western Kenya. Stove temperature data was recorded every five minutes using temperature sensors [38] (Geocene Dots) placed a standardized distance of 15 cm away from the centre of the flame on wood, charcoal and LPG stoves. Geocene Dots remained on the primary stoves used in these households for several months (November 2019-June 2020) prior to COVID-19 and during the community lockdown period. Temperature data was dichotomized into ‘stove on’ or ‘stove off’ based on a machine learning algorithm developed by Geocene [38]. The SUM data analysis provided an objective comparison of cooking patterns from full 6 kg cylinder LPG users relative to the PAYG LPG users in Kenya.

### 2.6 Data sharing

The anonymized PayGo Energy customer database was stored on a secure, cloud-based server hosted on the Google Cloud Platform and shared securely with University of Liverpool using DatAnywhere (Varonis Systems, New York City, USA). The interview recordings were securely shared with Liverpool using SharePoint. All statistical analyses were completed in R version 3.5.1 [42]. Ethical approval for this study was obtained from the University of Liverpool Central Ethics Committee, United Kingdom.

## 3 Results

### 3.1 Study population

Over 100,000 (n = 135,353) days of LPG customer data on PAYG LPG usage from 426 PayGo Energy customers (415 residential (97%) and 11 commercial (3%)) from January 2018 – June 2020 were analysed. Customers were primarily recruited in two separate ‘waves’ in 2017 and 2019. Thus, the distribution of months of smart meter data available per customer was bimodal with peaks at 8 months and 34 months (range: 1–41 months) (Table S6). There were 288 (68%) active customers as of June 30, 2020. Of the 136 (133 residential, 3 commercial) PayGo Energy account deactivations, 95% (n = 130) occurred in 2018 or 2019 - the most common reasons being moving away from the community (n = 41; 32%) or tampered/stolen equipment (n = 34; 26%) (Table S7). Only 13 customers deactivated their account (n = 6) or had no recorded PAYG LPG usage during the first three months (April-June 2020) of the COVID-19 lockdown (n = 7). Thus, 95% of PayGo Energy customers that were active in March 2020 continued using PAYG LPG during the lockdown.

Half (49%; n = 124) the PayGo Energy residential customers lived in households comprising one multi-purpose room (Table 1). Nearly half (44%; n = 110) of female heads of household had not completed more than primary level education, compared with 28% (n = 61) of male heads of household. A female was the main cook in four in five households (83%; n = 211), yet the primary decision maker for choice of cooking fuel in only 59% (n = 153) of households. This is, however, significantly higher (p = 0.05) than the proportion of female cooking fuel decision makers among households in the informal urban settlement not using PAYG LPG (47%, n = 49) (Table S3).

**Table 1:** Demographics and cooking preferences at the time of registration for pay-as-you-go LPG among residential customers (all data collected before COVID-19 lockdown) (N = 415).

Characteristic	N (%)
Cooking fuels used at time of registration with PayGo Energy (multiple responses allowed)	
LPG (Meko^1^)	113 (27%)
Kerosene	323 (78%)
Charcoal (Jiko^2^)	237 (57%)
Fuel stacking combinations at time of registration with PayGo Energy	
LPG only	57 (14%)
LPG + kerosene	20 (5%)
LPG + charcoal	12 (3%)
LPG + kerosene + charcoal	23 (5%)
Kerosene + charcoal	184 (46%)
Kerosene only	90 (23%)
Charcoal only	12 (3%)
Main lighting fuel	
Electricity/solar	169 (73%)
Kerosene, candles, other	73 (27%)
Number of home meals cooked/day	
2	34 (13%)
3	218 (82%)
4+	12 (5%)
Household size (no. of rooms)	
1	124 (49%)
2	68 (27%)
3+	59 (24%)
No. of household inhabitants	
1–2	58 (14%)
3–4	207 (48%)
5–6	122 (28%)
7+	41 (10%)
Female head level of education	
None	9 (4%)
Primary	101 (40%)
Secondary or university	143 (56%)
Male head level of education	
None	4 (2%)
Primary	57 (26%)
Secondary or university	160 (72%)
Sex of main cook	
Female	211 (83%)
Sex of cooking decision maker	
Female	153 (59%)

1. 6 kg cylinder with on top burner and ring top

2. Portable ceramic charcoal stove commonly used in Kenya

Note: Fuel combinations with n < 3 households not shown for brevity. Some demographic data only collected from a subset of PayGo Energy customers (number varies by variable) during certain years of enrolment. Numbers for certain variables do not sum up to overall sample size due to missing data.

One quarter (27%; n = 113) of households reported using a single burner LPG (Meko) stove prior to registering with PayGo Energy, with three-quarters (78%; n = 323) using a kerosene stove and over half (57%; n = 237) using a charcoal stove (Table 1). Of the 113 customers using LPG prior to registering with PayGo Energy, only half (n = 57) exclusively used LPG for cooking. Two PayGo Energy customers that were interviewed mentioned that they continued to use polluting cooking fuels when boiling some foods that require high gas consumption, such as cereals or beans, in order to save money. The overall prevalence of fuel stacking (use of multiple fuels) in the community was 60% (n = 239) prior to registering with PayGo Energy.

### 3.2 Pay-as-you-go LPG cooking patterns before COVID-19 lockdown

PayGo Energy residential customers used LPG for cooking an average of 1.4 cooking events/day (SD: 0.5) and four days/week (SD:1.3) (Table 2). Meals prepared using PAYG LPG lasted an average of<14 min (SD: 13.4). Average PAYG LPG consumption among residential customers over the analysis period (before COVID-19 lockdown) was 0.97 kg/capita/month (11.6 kg/capita/yr) (Table 2), which translated to 3.2 kg of LPG per household per month. Average PAYG LPG consumption among commercial customers was much higher (17.1 kg/month) (Table S9); all consumption patterns for the remainder of this analysis focus on residential customers, who make up the vast majority (97%) of PayGo Energy’s clientele.

**Table 2:** Pay-as-you-go LPG fuel usage and spending habits of residential customers before (January 2018-February 2020) and during (March 2020-June 2020) the COVID-19 lockdown **(N = 415)**.

Metric	Pre-Lockdown (all months)		Pre-Lockdown (January & February only)		Pre-Lockdown (June & July only)	
	Mean	SD	Mean	SD	Mean	SD
Kg of gas/capita/month	0.97	0.74	0.96	0.71	1.21	1.08
Cooking events per day	1.38	0.52	1.01	0.17	1.10	0.41
Number of days used/month	15.6	2.0	14.0	5.6	15.9	6.8
Number of days used/week	4.1	1.3	3.7	1.2	3.7	1.3
Kg of gas used per cooking event^1^	0.04	0.05	–	–	–	–
Cooking time per event (minutes)^2^	13.8	13.4	–	–	–	–
Days between payments (median (IQR))	8.0	[4.0, 22.0]	15.0	[6.0, 30.0]	10.0	[5.0, 30.0]
Total amount spent per month (KSh)	840	488	812	361	860	433
Number of payments/month	6.1	3.3	3.1	2.1	4.3	2.9
*LPG 6 kg cylinders under BCRM*: # of payments/month^5^	0.78	0.25	–	–	–	–
*Kerosene:* # of payments/month^3^	17.4	13.7	–	–	–	–
*Charcoal:* # of payments/month^3^	22.1	12.3	–	–	–	–
Single payment amount (KSh)	220	212	225	265	184	211
Kg of LPG credits purchased per payment	1.3	1.2	1.3	1.2	1.1	1.0
*LPG 6 kg cylinders under BCRM:* Single payment amount (KSh)^3^	988	25	–	–	–	–
*Kerosene:* Single payment amount (KSh)^3^	275	425	–	–	–	–
*Charcoal:* Single payment amount (KSh)^3^	156	315	–	–	–	–

1. Kg of gas used presented from a subset (n = 232; 53%) of customers using cylinder smart meters, which records consumption to the nearest 0.001 kg. Usage data from using Goldcard meters (which is rounded to the nearest 0.2 kg) was excluded for accuracy.

2. Data only available from a subset (n = 201; 46%) of customers using cylinder smart meters

3. Cross-sectional self-report survey data obtained only at time of customer registration with PayGo Energy (2017)

**Fig. 1:**
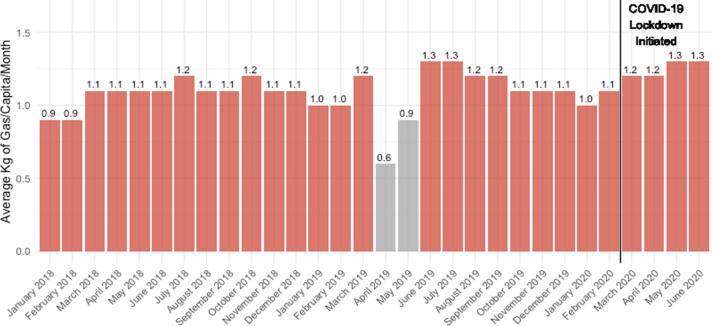
Average kilograms of LPG consumed per month per capita among pay-as-you-go LPG residential customers. Note: greyed bars in April-May 2019 reflect a time period when PayGo Energy updated their LPG metering technology from Goldcard meters to cylinder smart meters and replaced the equipment in several households; data during these months does not reflect true consumption.

To assess seasonal differences in LPG usage patterns, PAYG LPG consumption was examined exclusively in two months during the Kenyan hot, dry season (January, February) and the cooler, dry season (June, July) in 2018/2019 (prior to COVID-19 lockdown) (Table 2). Consumption was an average of 0.25 kg/capita/month lower in Kenyan hot, dry season (January-February: 0.96 kg/capita/month) compared with the cooler, dry season (June-July: 1.21 kg/capita/month) (Fig. 1). The potential effect of seasonal income fluctuations was documented in a PayGo Energy customer interview:

“Money to top up can be hard to find but you understand that it is just for a season before things revert back to normal.”

### 3.3 Pay-as-you-go LPG spending patterns

During the analysis period, customers spent an average of 840 Kenyan Shilling (KSh)/ US$7.69 (SD: 488) on PAYG LPG per month, with average single mobile money payments of $220 KSh/ US$2.01 (SD: 212) (Table 2). The mean mobile money payment amounts (220 KSh) were comparable to the average cost of kerosene in the community (275 Ksh) and more than the cost of single charcoal (156 KSh) in 2017. As PayGo Energy charged roughly 180 KSh/ US$1.66 per kilogram of LPG throughout 2018–2020, the mean mobile money payment translated to an average of 1.3 kg (SD: 1.2) of LPG credits purchased (approximately one-quarter of a typical 6 kg gas cylinder).

### 3.4 Pay-as-you-go LPG cooking patterns during COVID-19 lockdown

As the months of PAYG LPG customer data available during COVID-19 lockdown spanned March-June 2020, smart meter data from 2018 and 2019 was restricted to the same months to control for seasonal differences when assessing the impact of COVID-19 lockdown on PAYG LPG cooking behaviours. During lockdown, the average length of a cooking event decreased slightly (from 14.4 min to 13.5 min).

**Fig. 2:**
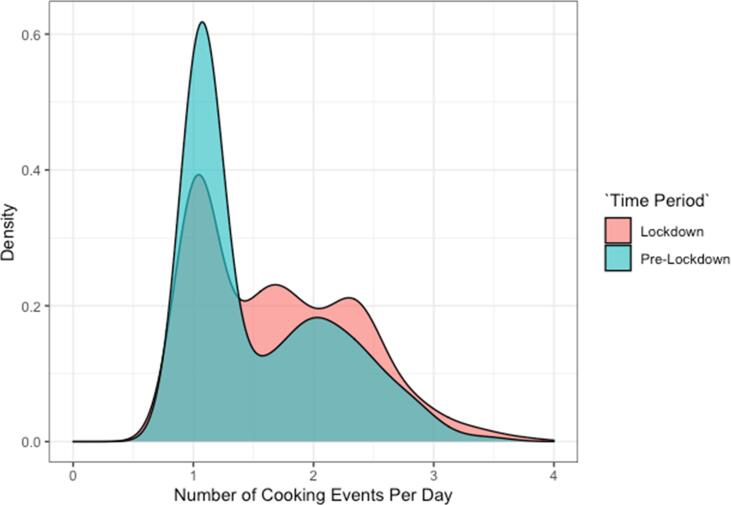
Number of cooking events per day using pay-as-you-go LPG stove before (‘Pre-Lockdown) and during COVID-19 community lockdown (‘Lockdown’).

However, the average number of daily cooking events during lockdown (1.72) was 60% higher than that of the same months in 2018/2019 (1.07) (Fig. 2). Further, the mean number of days per week using PAYG LPG increased from 4.3 (pre-lockdown) to 5.0 (lockdown) (p < 0.001). This resulted in an overall increase in PAYG LPG consumption during lockdown, from 1.12 kg/capita/month (SD: 0.75) to 1.25 kg/capita/month (SD: 1.01). Five out of six residential customers interviewed affirmed that their use of PAYG LPG during the lockdown increased due to having to prepare lunch while their children were home from school; children ordinarily eat their lunch at school.

### 3.5 Pay-as-you-go LPG spending patterns during COVID-19 lockdown

**Fig. 3:**
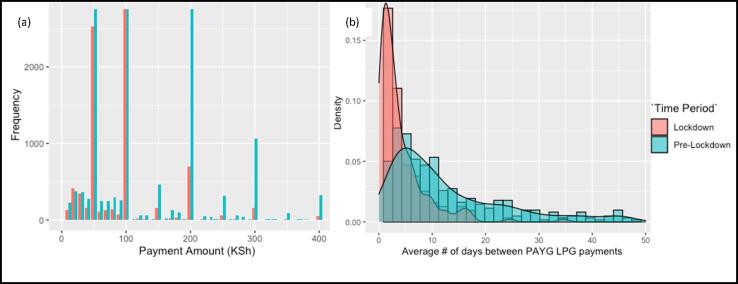
(a) Single mobile money payments made for pay-as-you-go LPG before (‘Pre-Lockdown’) and during COVID-19 community lockdown (‘Lockdown’) (b) Number of days between subsequent PAYG LPG payments before (‘Pre-Lockdown) and during COVID-19 community lockdown (‘Lockdown’).

Among households using PAYG LPG during the lockdown, the average single payment amount significantly dropped by nearly 50% (from 336 to 179 KSh/ US$3.08 to US$1.64) (p < 0.001) compared with payments made during March-June of 2018/2019 (Fig. 3a). This resulted in the mean amount of LPG purchased per payment shifting from 2 kg (pre-lockdown) to just over 1 kg (lockdown) (Table 3). Lower payments during lockdown (Fig. 3a) were offset by a 67% increase in median payment frequency - from 4.6 payments/month (SD: 3.2) to 7.7 (SD: 1.9) payments/month during lockdown (Table 3). This resulted in mobile money payments being made every 4 days (IQR:[2.5, 9.0]) during COVID-19 lockdown, compared with once every 8 days (IQR:[4.0, 22.0]) pre-lockdown (Fig. 3b). This change in payment patterns results in an insignificant decrease (p = 0.29) in total PAYG LPG monthly expenditure (867 Ksh/ $7.94 USD (pre-lockdown) to 816 Ksh/ $7.47 USD (lockdown)).

**Table 3:** Pay-as-you-go LPG fuel usage and spending habits of residential customers active during the COVID-19 lockdown (N = 298).

Metric	Pre-Lockdown (all months 2018/2019)		Pre-Lockdown (March-June 2018/2019)^1^		Lockdown (March-June 2020)		95% CI for difference^2^	p-value^3^
	Mean	SD	Mean	SD	Mean	SD		
Kg of gas/capita/month	0.98	0.30	1.12	0.75	1.22	1.01	[-0.02, 0.26]	0.10
Cooking events per day	1.42	0.59	1.07	0.23	1.72	0.65	[0.57, 0.73]	<0.001*
Cooking time per event (minutes)^4^	13.8	13.4	14.4	13.9	13.5	13.0	[-3.2,0.4]	0.12
Number of days used/month	14.8	5.0	16.5	5.8	19.0	6.8	[1.7, 3.4]	<0.001*
Number of days used/week	4.1	1.3	4.3	1.3	5.0	1.5	[0.5, 0.9]	<0.001*
Days between payments (median (IQR))	8.0	[4], [23]	8.0	[4.0, 22.0]	4.0	[2.5, 9.0]	[-4.0, −2.0]	<0.001*
Single payment amount (KSh)	220	212	336	286	179	189	[-158,-96]	<0.001*
Kg of LPG credits purchased per payment	1.3	1.2	2.0	2.6	1.1	1.1	[-1.1, −0.4]	<0.001*
Amount spent per month (KSh)	840	488	867	469	816	510	[-99, 20]	0.29
Number of payments/month	6.1	3.3	4.6	3.2	7.7	1.9	[0.4, 1.5]	0.001*
*LPG 6 kg cylinders under BCRM*: # of payments/month^5^	0.78	0.25	–	–	–	–	–	–
*Kerosene:* # of payments/month^4^	17.4	13.7	–	–	–	–	–	–
*Charcoal:* # of payments/month^4^	22.1	12.3	–	–	–	–	–	–
*LPG 6 kg cylinders under BCRM:* Single payment amount (KSh)^4^	988	25	–	–	–	–	–	–
*Kerosene:* Single payment amount (KSh)^4^	275	425	–	–	–	–	–	–
*Charcoal:* Single payment amount (KSh)^4^	156	315	–	–	–	–	–	–

1. Restricted to months March-June to control for season variation in cooking patterns when comparing to cooking metrics during COVID-19 lockdown

2. 95% confidence interval from paired T-test (or Wilcoxon signed-rank test for right skewed data) for difference between pre-lockdown (n = 298) and lockdown cooking patterns

3. P-value from paired *t*-test (or Wilcoxon signed-rank test for right skewed data) for difference between pre-lockdown (n = 298) and lockdown cooking patterns

4. Data only available from a subset (n = 201; 46%) of customers using cylinder smart meters. Cooking time data unavailable for March-June in 2018/2019 as households did not yet have cylinder smart meters.

5. Cross-sectional self-reported survey data obtained only at time of customer registration with PayGo Energy

*Significant at alpha = 0.05 level

### 3.6 Pay-as-you-go LPG consumption by socioeconomic characteristics

Per capita consumption among customers that purchased full LPG refills under the BCRM before registering for PAYG LPG substantially increased by 0.35 kg/month during the lockdown (from 0.88 (SD: 0.17) to 1.23 (SD: 0.05)) (Table 4). PAYG LPG monthly per capita consumption increased by less than half that of previous LPG users (0.15 kg/capita/month) among households that had not used LPG before PAYG LPG registration during the lockdown (from 1.12 (SD: 0.24) to 1.27 (SD: 0.03). PAYG LPG per capita consumption increased by approximately 0.4 kg/capita/month during the lockdown among households where the head female was employed in the formal or informal sector, while only slightly increasing (0.1 kg/capita/month) among those employed in casual jobs and decreasing substantially among those unemployed before lockdown (-0.88 kg/capita/month) (Table 4). Average PAYG LPG monthly consumption also increased among households with three or more family members, while decreasing among those with one or two family members. The reason for this dichotomy is likely because of the need to prepare lunch for children home from school during lockdown.

**Table 4:** Monthly Per Capita pay-as-you-go LPG consumption by pre-registration fuel choice and occupation (N = 277).

	N	Monthly Kg Per Capita Usage (Mean (SD))		Cooking Time Per Day (Minutes)* (Mean (SD))		Single payment amount (KSh) (Mean (SD))		Total Spent per month (KSh) (Mean (SD))	
		Pre-Lockdown (March -June 2018 & 2019)	Lockdown (March - June 2020)	Pre-Lockdown (All months 2018 & 2019)	Lockdown (March – June 2020)	Pre-Lockdown (March -June 2018 & 2019)	Lockdown (March – June 2020)	Pre-Lockdown (March -June 2018 & 2019)	Lockdown (March – June 2020)
LPG user prior to PAYG LPG									
Yes	91	0.88 (0.17)	1.23 (0.05)	62 (26)	65 (30)	304 (2 8 5)	180 (2 3 2)	820 (77)	793 (42)
No	186	1.12 (0.24)	1.27 (0.03)	80 (72)	81 (65)	346 (2 9 7)	175 (1 8 0)	870 (50)	832 (33)
Primary fuels prior to PAYG LPG									
Kerosene only	64	1.14 (0.26)	1.32 (0.06)	64 (32)	67 (34)	331 (3 0 7)	159 (1 6 4)	789 (63)	858 (46)
Charcoal only	11	1.25 (0.90)	1.34 (0.10)	194 (1 5 4)	158 (1 5 7)	238 (1 5 0)	132 (89)	404 (1 6 2)	576 (1 2 3)
Kero. + charcoal	111	1.11 (0.24)	1.22 (0.05)	68 (39)	70 (34)	351 (2 9 9)	187 (1 9 5)	892 (59)	828 (25)
LPG only	50	1.30 (0.34)	1.33 (0.07)	61 (22)	66 (26)	351 (3 8 8)	196 (2 8 2)	770 (1 7 4)	770 (48)
LPG + Kerosene	18	0.74 (0.20)	1.12 (0.07)	65 (37)	52 (35)	180 (1 0 5)	146 (1 8 2)	707 (1 7 8)	752 (43)
LPG + Charcoal	8	1.03 (0.35)	1.39 (0.17)	81 (15)	73 (42)	218 (1 2 2)	165 (1 5 6)	1077 (2 1 6)	1160 (46)
LPG + Charcoal+ Kerosene	15	0.76 (0.15)	0.98 (0.08)	45 (19)	46 (25)	354 (3 0 9)	183 (1 2 3)	857 (1 2 1)	742 (82)
Occupation (female)									
Formal job	7	1.12 (0.26)	1.52 (0.11)	140 (11)	132 (25)	207 (1 0 9)	141 (66)	1068 (90)	1142 (49)
Informal sector job	23	1.03 (0.29)	1.39 (0.08)	70 (46)	61 (35)	390 (3 4 3)	248 (2 7 4)	832 (61)	941 (49)
Day labourer	13	0.99 (0.24)	1.09 (0.1)	102 (40)	83 (25)	263 (3 5 7)	166 (1 1 8)	779 (1 9 3)	815 (1 0 8)
Unemployed	2	1.50 (0.63)	0.62 (0.08)	–	–	560 --	100 --	1516 --	1110 --
No. people in home									
1–2	58	2.22 (0.47)	2.14 (0.25)	47 (24)	49 (38)	331 (3 0 7)	159 (1 9 5)	843 (76)	706 (63)
3–4	201	1.09 (0.24)	1.29 (0.02)	69 (44)	71 (38)	347 (3 1 5)	183 (2 3 0)	821 (56)	783 (40)
5–6	118	0.78 (0.16)	0.91 (0.03)	81 (59)	82 (64)	310 (2 4 1)	168 (1 3 6)	840 (41)	854 (56)
7+	41	0.56 (0.13)	0.68 (0.06)	119 (1 1 4)	113 (94)	361 (3 3 2)	176 (1 3 8)	981 (80)	1079 (36)

*Cooking time per day only available for a subset (n = 201; 46%) of PayGo Energy customers with cylinder smart meters. Data provided for all months in 2018/2019 as cylinder smart meters weren’t installed until after June 2019.

Note: some demographic data only collected from a subset of customers during certain years of enrollment by PayGo Energy (sample size varies by variable). Numbers for certain variables do not sum up to overall sample size due to missing data. Male occupation not presented due to low sample size.

Similarly, monthly expenditure on PAYG LPG was higher during COVID-19 lockdown among households with the female household head employed in formal or informal sectors compared with a decrease in expenditure in households where the female head was unemployed or worked as a day labourer. Total monthly amount spent on PAYG LPG increased among households with five or more family members, while decreasing among families with four members or less (Table 4). Single payment amounts decreased by ~ 50% among households not previously purchasing full cylinder LPG while decreasing only 40% among households using LPG prior to registering with PayGo Energy (Table 4). Notably, average monthly expenditure on PAYG LPG during lockdown increased by the largest margin among households previously using charcoal before registering with PayGo Energy. One interviewed customer attributed their higher use of PAYG LPG to higher prices of polluting fuels in the community:

“Gas is more economical compared to kerosene or charcoal […] gas prices are affordable and [PAYG LPG] is sustaining us now.”

### 3.7 Changes in pay-as-you-go LPG consumption with user experience

PAYG LPG monthly per capita consumption gradually increased overtime among PayGo Energy customers (Table 5). In the first 6 months since registering with PayGo Energy, average customer consumption was 0.76 kg/capita/month; monthly per capita consumption increased to 0.96 kg/capita month after households had been PAYG LPG customers for at least 6 months. The increase in monthly consumption was complemented by an increase in monthly expenditure by nearly 100 KSh between the first and second year as a PAYG LPG customer, from an average of 735 Ksh ($US6.75) spent per month in months 1–6 to 820 KSh (US$7.53) in months 19–24.

**Table 5:** Long-term consumption patterns by length of time as a pay-as-you-go LPG customer (among households that registered with PayGo Energy in 2017) (N = 207*).

Number of months since PAYG LPG account activation	N	Kg of gas/ capita/month		Number of days used/month		Cooking events per day		Amount spent per month (KSh)	
		Mean	SD	Mean	SD	Mean	SD	Mean	SD
1–6^1^	125	0.76	0.63	12.4	5.3	1.03	0.11	735	437
7–12	181	0.96	0.67	14.3	5.6	1.05	0.04	791	458
13–18	207	0.98	0.72	13.9	5.7	1.05	0.08	802	526
19–24	196	1.09	0.99	12.9	5.6	1.07	0.08	820	553

*This sample size includes only customers recruited in ‘Wave 1′ (i.e. in 2017) to examine cooking patterns unimpacted by COVID-19 lockdown. This example excludes households that deactivated their account within two years of registration to examine long-term cooking behaviours. Smart meter data from January and February were excluded from this analysis to control for seasonal cooking pattern differences confounding the relationship.

1. The sample size for months 1–6 is smaller as 82 households recruited in Wave 1 prior to September 2017 were not initially installed with a smart meter, so no data is available. A sensitivity analysis with these 82 households excluded revealed no significant changes in long-term consumption patterns.

Customers that did not deactivate their PayGo Energy account in 2018/2019 had a higher proportion (61%) of female household members in charge of cooking fuel decisions than males, compared with households that deactivated their PAYG LPG account during this period (48%) (Table 6). Additionally, the female household head was significantly more likely (p = 0.04) to be employed in a formal sector job and less likely to be unemployed among households keeping their PAYG LPG account active, compared with customers that deactivated their PAYG LPG account in 2018/2019. There was no association between (Meko) LPG stove use prior to registering with PAYG LPG and customers maintaining or deactivating their PayGo Energy account during the study period.

**Table 6:** Factors associated with pay-as-you-go LPG account deactivations in 2018/2019 (before COVID-19 lockdown) (N = 247^1^).

	Active Account (N = 186)	Deactivated Account (N = 61)	p-value (χ^2^ test)
Sex of cooking fuel decision maker			0.12
Female	105 (61%)	29 (48%)	
Male	67 (39%)	31 (52%)	
Female household head education			0.18
None	7 (4%)	0	
Primary	73 (39%)	19 (31%)	
Secondary/university	90 (48%)	37 (61%)	
Female occupation			0.04*
Formal job	8 (14%)	1 (6%)	
Informal sector job	28 (50%)	6 (35%)	
Day labor/casual job	17 (30%)	5 (29%)	
Unemployed	3 (5%)	5 (29%)	
LPG user prior to PAYG LPG			1.0
Yes	23 (13%)	8 (13%)	
No	157 (87%)	53 (87%)	

1. This sample size includes only customers recruited in ‘Wave 1′ (i.e. in 2017) to ensure that PayGo Energy account deactivations during the first two years of registration were not impacted by COVID-19 lockdown. Some variables don’t add up to total sample size because of missing data.

*Significant at alpha = 0.05 level

### 3.8 Perceived benefits of pay-as-you-go LPG versus conventional full cylinder LPG and kerosene

PAYG LPG was revealed to have a number of benefits compared with purchasing a pre-set 6 kg cylinder of LPG or kerosene. Benefits reported by seven interviewed participants included fuel affordability, safety from burns/gas explosions, time savings, ease of fuel access and user-friendliness of the smart meters. The ability to make small, regular payments was critical to participants being able to cook with LPG, who were reassured to know that they could still prepare meals for their children with only small amounts of money at their disposal:

“Because even if you have the lowest amount of money, you will still be able to cook.”

“It is okay because if I have even 20 KSh, I can refill [make a mobile money payment] and finish cooking.”

For some customers who already had access to conventional cylinder LPG before registering with PayGo Energy, PAYG LPG often serves as a backup fuel source when they may have less cash on hand:

“[PAYG LPG] has helped me in such a way that if I don’t have enough money to fill the bigger cylinder, I can get PayGo.”

The value for money and efficiency in terms of the amount of meals that could be prepared was another commonly reported benefit discussed by PayGo Energy customers when interviewed:

“*PayGo is very economical because when I was buying kerosene, I would pay 100 KSh and it would not be enough for my cooking. I could not boil water, use for bathing and cook supper with [a single purchase of] kerosene*…*I would not have any [fuel] left to cook breakfast in the morning. Now when I refill gas [using PAYG LPG] for 100 KSh, I use it for all those things including breakfast the next day without straining.”*

Multiple first time LPG users also favored the technology itself. They enjoyed the efficiency of cooking of the double-burner LPG stove, which they reported saves time by enabling them to cook dishes (e.g. ugali and vegetables) simultaneously on each burner. PAYG LPG was also considered to be more convenient because participants did not have to carry the cylinder to the LPG retailer, nor did they have to buy matchsticks to start the flame. Another interviewee pointed out the benefits of using the mobile money system (M−Pesa) for purchasing LPG credits, as other community members paying for cooking fuels via a ‘card system’ are sometimes unable to cook if the card is misplaced.

Increased safety was also a reported advantage of PAYG LPG as several customers interviewed were worried about gas explosions and risks of their children being burned if purchasing full cylinder LPG. Many interviewees stated they had greater peace of mind cooking with PAYG LPG because “when there is a leakage [PayGo Energy customer support] can detect from the main office and shut off [the gas].” One interviewee indicated that they previously refrained from buying full cylinder 6 kg LPG (and used kerosene instead) because a friend in the community suffered a burn when cooking with gas; had it not been for the safety of smart meter technology of PAYG LPG, this customer would not be cooking with LPG until their children were older and could be trusted to keep their distance from the cookstove and cylinder.

LPG was viewed as an aspirational fuel for the interviewees: “*I never had the hope of one day owning an LPG [stove] in my home*”. Another customer stated: “*And by the way, I have seven children and I [am able to use] LPG*” because of PAYG LPG. Indeed, increased monthly consumption and expenditure of PAYG LPG among households with seven or more members during lockdown (Table 4) suggests that LPG was able to accommodate higher cooking needs due to several children being home from school.

### 3.9 Stove use monitoring data in Eldoret, Kenya

Study households in Eldoret, Kenya had statistically significantly higher SES than PayGo Energy customers in Mukuru kwa Reuben by all factors examined (education, occupation, household size, and electricity access for lighting) (Table S4). Despite higher SES in Eldoret, PAYG LPG companies have not yet penetrated *peri*-urban communities in Kenya due to higher infrastructure costs of distributing outside large cities. Thus, LPG in Eldoret is currently only available through full filled cylinder purchase (6 kg cylinders are the most common size).

**Fig. 4:**
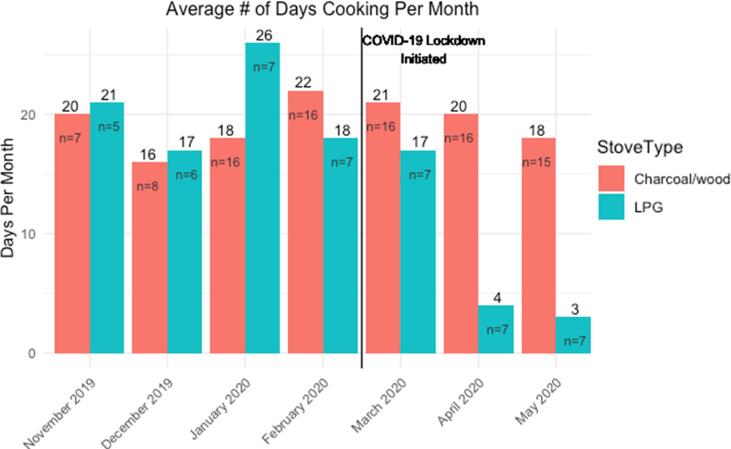
Average number of days per month cooking with LPG versus traditional stoves in Eldoret, Western Kenya before and during a COVID-19 lockdown measured with stove use monitors.

Among seven households from Eldoret with available SUM data and primarily cooking with LPG, the number of days/month using the cooking fuel declined by 75% during the COVID-19 lockdown, from an average of 17 days in March 2020 to four days or less in April-May 2020 (Fig. 4). In contrast, households primarily cooking with charcoal/wood (n = 16) continued to use these fuels consistently during lockdown, approximately 20 days per month (Fig. 4).

**Fig. 5:**
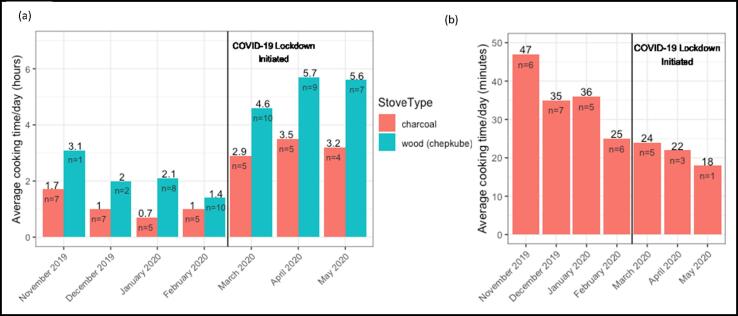
Average hours per day per month cooking on (a) wood and charcoal stoves and (b) LPG stoves in Eldoret, Kenya before and during a COVID-19 lockdown.

The average duration of cooking per day (hours) using charcoal and wood stoves increased by 200–300% in Eldoret during lockdown (e.g. wood stove daily cooking time increased from 2 to 5.5 h/day) (Fig. 5a). Conversely, among those who continued to use LPG (n = 5), daily cooking time decreased slightly during lockdown (Fig. 5b).

## 4 Discussion

This study is the first to report long-term cooking and spending patterns in East Africa using PAYG LPG smart meter data. This analysis also uniquely assessed use of PAYG LPG in the context of economic instability introduced by COVID-19 control measures. Among 415 PAYG LPG residential customers, annual LPG per capita consumption was 11.6 kg/capita/year, below that of estimated average consumption among *peri*-urban dwellers (12.8 kg/capita/year) and urban dwellers (18.7 kg/capita/year) [43]. The mean payment made by PayGo Energy customers (220 KSh), which equated to 1.3 kg of LPG (Table 3), was similar to the typical amount spent for kerosene in the community (275 KSh), and approximately one-fourth of what one would typically pay for a full 6 kg cylinder of LPG (988 KSh), suggesting a preference to pay for gas in smaller, more frequent increments than required for the full LPG cylinder. On a monthly basis, the average amount spent by PAYG LPG residential customers (840 Ksh/month (US$7.69)) was similar to the paid amount reported by purchasers of LPG cylinder refills under the BCRM in the same community (850 Ksh/month (US$7.83)) [27]. Similar average monthly expenditures among PAYG LPG customers despite lower usage of PAYG LPG (4 days/week) (Table 2) compared to self-reported use of LPG under the BCRM (6.3 days/week) [27] is due to a ~ 7.1% fuel surcharge by PayGo Energy to cover equipment installation and delivery fees [28], [44].

Additional to the flexible payment scheme offered by PAYG LPG, customer interviews revealed that increased safety from burns/gas explosions, time savings, ability to prepare multiple dishes simultaneously using the provided double-burner stove and fuel delivery were also key features enjoyed by households. The participants indicated that they could carry out a higher amount of cooking/bathing tasks with PAYG LPG compared with the same amount spent on kerosene. While four of the six interviewed residential households used PAYG LPG to meet all of their cooking needs (with the exception of electricity (e.g. electric jug) to heat up water for bathing), two interviewed customers indicated that they continued to cook with kerosene or charcoal alongside PAYG LPG. These customers used polluting fuels for boiling foods such as cereals or beans to save money, as they believe they would not be able to afford the amount of gas required to prepare food items that required a prolonged cooking time.

Cooking fuel stacking (concurrent use of multiple clean and polluting fuels) by the study population may be a primary cause of lower annual per capita consumption among PAYG LPG users relative to those purchasing the full LPG cylinder. Fuel stacking was high (60%) among LPG users buying full cylinder refills prior to using PAYG LPG (Table 1). As PAYG LPG consumption during the lockdown notably decreased among those employed in casual jobs or unemployed (Table 4), the inability to afford exclusive use of LPG under a PAYG scheme may lead to stacking with polluting fuels for some resource-poor households. Nonetheless, five of the seven PayGo Energy customers interviewed indicated that they would be cooking kerosene had they not had access to PAYG LPG. Thus, the ability to pay in smaller payments did provide families that aspired to cook with LPG access to the fuel.

### 4.1 Impacts of COVID-19 lockdown on pay-as-you-go LPG cooking behaviours

Despite financial hardships imposed by the COVID-19 lockdown (95% of households reported decreases in household income during lockdown [27]), an increase in monthly per capita PAYG LPG consumption was observed among residential customers (from 1.12 kg/capita/month pre-lockdown to 1.22 kg/capita/month during lockdown) (Table 3), and 95% (n = 288) of active PayGo Energy customers continued to use PAYG LPG during the COVID-19 lockdown (March-June 2020). A rise in LPG consumption during lockdown was likely the result of children staying at home during the day due to school closures (children consumed lunch at home when they would ordinarily eat this meal at school); children being home from school was reported by five out of six interviewed PayGo Energy residential customers as the reason for their increase in PAYG LPG usage.

While only 5% of PayGo Energy customers discontinued their use of PAYG LPG during the COVID-19 lockdown, a previous longitudinal survey conducted in the same community revealed that 27% of households using LPG under the BCRM as a primary cooking fuel reverted to purchasing kerosene (14%) or gathering wood for free (13%) during lockdown [27]. Further, the same study found that four (67%) of six households using full cylinder LPG with a greater number of people to cook for during lockdown switched to wood for cooking. This proportion is much greater than the 22% (10 out of 44) of households with no change in the number of household members during lockdown that switched from full cylinder LPG to wood or kerosene for cooking [27]. Moreover, one-third (n = 2 of 6) of households in Mukuru kwa Reuben cooking with kerosene that had to cook for more household inhabitants during lockdown switched to wood (which they could obtain for free on the side of the road), compared with 8% (n = 8 of 97) of kerosene users that reported no changes in family size switching to wood or charcoal for cooking. Thus, increases in LPG consumption among PayGo Energy customers that occurred by having to cook more frequently for more individuals may not have occurred had they been purchasing LPG under the BCRM.

The juxtaposition of LPG cooking habits between PayGo Energy customers and full cylinder LPG users within the same community supports the utility of the flexible payment schedule (e.g. smaller amounts more frequently) provided by PAYG LPG, which potentially allowed customers to maintain use of LPG despite income declines during COVID-19 lockdown (88% of community members reported complete cessation of income during lockdown [27]). Access to PAYG LPG may have therefore minimised the rise in community-level household air pollution emissions from increased use of wood/kerosene for cooking. This is particularly significant considering that three-quarters of PayGo Energy customers were first time LPG users (Table 1).

It is unlikely that higher SES among PayGo Energy customers accounted for greater LPG usage during the lockdown compared with full cylinder LPG users; while a sensitivity analysis revealed that PayGo Energy customers had slightly larger household size and higher prevalence of LPG usage under the BCRM (48%) (at the time of registration for PayGo Energy), compared with the community-level average (37%), female occupation and household education levels were similar between both groups (Table S3). Moreover, individuals living in Eldoret that were more affluent than PayGo Energy customers in Mukuru kwa Reuben (Table S4) reduced their use of full cylinder refill LPG (days/month) by 75%, providing further evidence of the value of PAYG LPG in preventing a reversion to polluting cooking fuels during lockdown.

Modifications to cooking patterns among PAYG LPG users were complemented by alterations in the rate of mobile money payments. The median number of days between subsequent payments was reduced by half (from once every eight days pre-lockdown to once every four days during lockdown) (Fig. 3b). However, the average payment amount decreased by nearly half (from 336 Kenyan Shillings (KSh)/US$3.08 (pre-lockdown) to 179 KSh/US$1.64 (lockdown)) (Table 3). Despite a higher volume of payments in smaller increments during the lockdown because of declines in household income, customers’ total monthly PAYG LPG expenditure remained relatively constant (867 Ksh/ $7.94 USD (pre-lockdown) to 816 Ksh/ $7.47 USD (lockdown)).

### 4.2 Socioeconomic factors associated with changing cooking and payment behaviors during COVID-19 lockdown

The increase in PAYG LPG per capita consumption and monthly expenditure during lockdown was driven by households with a higher number of family members (e.g. children staying home from school); households with at least three family members increased their PAYG LPG consumption during lockdown, while households with two people or less reduced their consumption. Customers that previously used LPG prior to registering with PayGo Energy increased their PAYG LPG monthly consumption by twice as much (0.35 kg/capita/month) as customers that had not previously used full cylinder LPG (0.15 kg/capita/month) (Table 4). Higher SES among previous LPG users may partially explain these observed differences; female head of households with jobs in the formal or informal sector increased their PAYG LPG monthly spending during lockdown by 80–90 KSh/month, while spending among those with casual jobs increased by 35 KSh and decreased among those that were unemployed. Monthly spending on PAYG LPG increased most substantially among households only cooking with charcoal or both full cylinder LPG and charcoal prior to the lockdown (Table 4); this may be due to a national ban on the sale of charcoal that was enacted in 2019 to reduce deforestation [45], which drove up the price of charcoal in the community.

### 4.3 Seasonality

When restricting smart meter data from 2018 to 2019 (pre-lockdown) to four specific months in hot or cool season, monthly PAYG LPG consumption and expenditure was lower in January and February relative to June and July (Table 2). Household incomes are typically lower during the start of the year in this community due to higher expenditure over the December holiday period and school-related expenses that come due in January. These seasonal LPG usage fluctuations, however, are less extreme than what has been observed in India, with LPG cylinder refill sales falling 10% lower in ‘non-cropping’ season than during cropping/harvest seasons when families typically have higher income [18]. As PAYG LPG allows consumers to adjust their payment amount based on their financial situation, it may be useful to expand this model to other countries where income changes seasonally.

### 4.4 Customer experience and retention

Average PAYG LPG monthly per capita consumption also gradually increased over time, from 0.76 kg/capita/month (SD: 0.63) in months 1–6 to 1.09 kg/capita/month in months 19–24 (Table 5). The reason for the increase is partially a result of lower consumers of PAYG LPG deactivating their account in 2018 or 2019. Households that did not deactivate their PayGo Energy account during the study period had a higher percentage of female cooking fuel decision-makers and female household heads employed in the formal or informal sector compared with households that deactivated their account (Table 6). This information may be useful for future commercial companies seeking to target the ‘early adopters’ of PAYG LPG technology and ensure continuity of their customer base.

### 4.5 Female empowerment

Prior to registering for PAYG LPG, females in households already using LPG 6 kg cylinders decided which cooking fuels would be used in their home at a 20% higher rate (77%) than households not using LPG (55%). Among households registering to use PAYG LPG, the proportion of females making decisions about cooking fuel purchases (59%) was significantly higher than the proportion in the community that did not register for PAYG LPG (47%) (Table S3). Further, female participants working in the informal sector or working as a day labourer used their PAYG LPG stove significantly less than female participants in formal employment both before and during the lockdown (Table 3). Lastly, the proportion of households with female cooking fuel decision-makers was higher among households that did not deactivate their PayGo Energy account prior to the pandemic (61%) compared with households that deactivated their account (48%).

Thus, policies that foster fiscal and social empowerment of women may have important co-benefits in fostering the transition to clean cooking [46]. These policies are especially important during COVID-19, as female informal sector workers were among the first to lose their job and livelihoods during a lockdown [47], [48], potentially resulting in additional health burden (e.g. poor mental health) [49], [50].

### 4.6 Strengths and limitations

With a sample of 415 residential PayGo Energy customers, this study was powered to examined cooking behaviours before and during a nationwide lockdown in Kenya. As customers were recruited primarily through door-to-door advertising, PayGo Energy staff members identifying a new customer and customer referrals, PAYG LPG customers may not be representative of the general population of the informal settlement. However, a comparison of available SES characteristics of PAYG LPG households with that of a random sample from the same community revealed no significant differences in female occupation and household education levels (Table S3). Further, as individuals from Eldoret had a higher SES than PayGo Energy customers living in Nairobi, a 75% decline in days per month using full cylinder LPG in Eldoret signals that household SES likely does not explain differences in LPG usage patterns between PAYG LPG and full cylinder LPG users in Mukuru kwa Reuben during lockdown.

As per capita LPG consumption varies according to the level of urbanicity [43], PAYG LPG usage in an informal urban settlement may not manifest in *peri*-urban or rural communities due to differences in availability of LPG refills (e.g. frequency of LPG cylinder deliveries), baseline familiarity with LPG among residents and sociocultural barriers [26], [51]. Moreover, home delivery of LPG refills in *peri*-urban and rural communities with lower population density may lead to higher fuel surcharges transferred to customers due to longer travel distances and more complex distribution logistics, influencing customer retention. Incorporation of predictive analytics by commercial companies to monitor the LPG supply chain may help improve the efficiency of delivery routes and therefore optimise distribution [32]. Issues related to infrastructure should be considered, as power outages and connectivity issues with mobile service providers may be a significant barrier in rural communities, which can hamper customers’ ability to use mobile money for LPG payments. Improved cellular infrastructure can help alleviate reliability concerns.

This study did not monitor cooking patterns among secondary cooking fuels used alongside PAYG LPG. If stove stacking persists among PAYG LPG customers, levels of household air pollution will likely remain above WHO guidelines [52], [53]. Fuel stacking can also impact the profitability of commercial PAYG LPG companies, as their business models are dependent on consistent LPG consumption due to low profit margins; thus, lower PAYG LPG usage due to stove stacking can disincentivize future market entry. Therefore, from both public health and financial perspectives, more research on stove stacking in the context of PAYG LPG is warranted.

## 5 Conclusion

### 5.1 Utility of pay-as-you-go LPG smart meter data

PAYG LPG smart meter data offers commercial companies the ability to measure customers’ consumption patterns in real time, without the need for external stove use monitoring. Smart meter data can be analysed to calculate cooking metrics for characterizing LPG usage and spending patterns over time, and may potentially be a useful input for carbon finance projects aiming to reduce climate-forcing emissions by increasing use of LPG for cooking. Smart meter data can further be combined with sociodemographic information obtained at time of customer enrolment to determine households most frequently using PAYG LPG to increase customer retention and predict when customers’ fuel will be depleted, informing inventory and staffing requirements. Improved operational efficiency by PAYG LPG commercial companies can translate to lower distribution costs, which can promote efficient scale up of operations.

### 5.2 Pay-as-you-go LPG can advance clean cooking

PAYG LPG was robust to consumers’ financial hardships induced by the COVID-19 control response by allowing for incremental fuel payments and ensuring a consistent fuel supply via home delivery. The quantitative and qualitative analyses in this paper also underscore how PAYG LPG addressed the cooking needs of families regarding safety, convenience, ease of payment and speed of cooking. By promoting sustained use of clean cooking in a resource-poor, urban community where individuals commonly work in the informal sector, living on daily wages and often experiencing seasonal income fluctuations, PAYG LPG is an encouraging consumer finance mechanism that can help achieve universal energy access (Sustainable Development Goal 7).

Author Statement

MS, EP, DP and MOK designed the study. MS derived the data analysis plan, managed, cleaned and analyzed all data, and wrote the first and final drafts of the paper. MOK supervised data collection and data storage. ES assisted with translation of customer interviews to English. EP and DP supervised the interpretation of the data. All co-authors assisted with data interpretation and reviewed the final manuscript.

## Declaration of Competing Interest

Mark O’Keefe is co-founder and Product Manager at *PayGo Energy*. His employment at *PayGo Energy* had no impact on interpretation of the data.
